# Investigation of the perceptual and cognitive asymmetry in the auditory system in patients with adolescent idiopathic scoliosis

**DOI:** 10.4102/sajp.v77i2.1583

**Published:** 2021-09-29

**Authors:** Burçin Akçay, Gonca İnanç, Ata Elvan, Metin Selmani, Mehmet A. Çakiroğlu, Ömer Akçali, İsmail S. Satoğlu, Adile Oniz, İbrahim E. Şimşek, Murat Ozgoren

**Affiliations:** 1 Department of Physiotherapy and Rehabilitation, Faculty of Health Sciences, Bandırma Onyedi Eylül University, Balıkesir, Turkey; 2 Faculty of Health Sciences, Near East University, Mersin, Turkey; 3 Department of Prosthesis-Orthosis, School of Physical Therapy and Rehabilitation, Dokuz Eylül University, Izmir, Turkey; 4 Physiotherapy Clinic, Izmir, Turkey; 5 Department of Therapy and Rehabilitation, İzmir Kavram Vocational School, Izmir, Turkey; 6 Department of Orthopaedics and Traumatology, School of Medicine, Dokuz Eylül University, Izmir, Turkey; 7 Department of Orthopaedics and Traumatology, Özel Sağlık Hastanesi, İzmir, Turkey; 8 Department of Biophysics, Faculty of Medicine, Near East University, Mersin, Turkey

**Keywords:** adolescent idiopathic scoliosis, dichotic listening paradigm, perceptual asymmetry, cognitive asymmetry, top-down process

## Abstract

**Background:**

Studies have shown that perceptual and cognitive asymmetries are present in the auditory system in patients with adolescent idiopathic scoliosis (AIS). The Dichotic Listening (DL) paradigm was formerly performed in non-forced (NF) conditions only, and no study has examined the conditions of attention to one ear.

**Objective:**

To investigate the perceptual and cognitive asymmetry in the auditory system in patients with AIS as well as the asymmetry changes according to the curvature characteristics of patients with AIS.

**Method:**

The DL paradigm was performed on 38 patients with AIS and 10 healthy individuals in all conditions (NF, Forced Right [FR], Forced Left [FL]).

**Results:**

In the NF and FL conditions, the mean number of correct responses for the left ear was significantly lower in patients with AIS than in healthy individuals (*p* < 0.05). The correct responses for the right ear in the NF condition, right and left ear in the FR condition, and right ear in the FL condition did not show a significant difference between the groups (*p* > 0.05). Also, there was no difference between patients with AIS with both functional 3-curve and 4-curve (*p* > 0.05).

**Conclusion:**

Our study indicates perceptual and cognitive asymmetry or lateralisation in the auditory system in patients with AIS. The asymmetry might be caused by the inability to direct their attention to the left ear, which is not affected by their curvature type. Further studies are needed to investigate perceptual and cognitive asymmetry behaviour models in the auditory system in patients with AIS.

**Clinical implications:**

Determination of perceptual and cognitive asymmetry in the auditory system may offer a new perspective on conservative treatment protocols for AIS patients. Besides, the DL paradigm can be easily used in patients with AIS as a non-invasive evaluation method in clinics.

## Introduction

Skeletal, muscular, postural, sensory-motor function, and neurological asymmetries may develop because of, or with scoliosis in patients with adolescent idiopathic scoliosis (AIS) (Burwell, Dangerfield & Freeman 2008; Lowe et al. 2000; Provencher, Wester & Gillingham 2003). Studies investigating neurological changes in AIS have tried to explain the aetiopathogenesis of AIS and have shown neurological asymmetries (Schlösser et al. 2014).

When cranium morphology is evaluated, the occipital contour is smaller in patients with AIS, while the left parietal contour is more prominent than the right (Chu et al. 2007; Yeung et al. 2007). In terms of brain morphology, cortical thickness differences and cerebellar volume changes are observed between both hemispheres in patients with AIS (Liu et al. 2008; Wang et al. 2012). These studies support the presence of hemispheric asymmetry with changes in brain morphology in patients with AIS. Considering the electroencephalography (EEG) studies evaluating the structures and functions of the brain, it is seen that there is no consensus in terms of changes in brain responsiveness or hemispheric differences (Dretakis et al. 1988; Petersen, Sahlstrand, & Sellden 1979; Pinchuk et al. 2012). Apart from EEG, the brain structures’ functions have been evaluated with the Dichotic Listening (DL) paradigm in AIS patients, and it is stated that perceptual and cognitive asymmetries in the auditory system are present (Enslein & Chan 1987; Goldberg et al. 1995).

**FIGURE 1:**
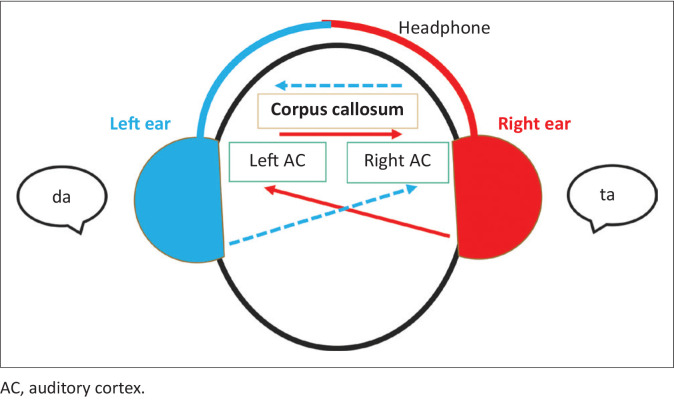
Doreen Kimura’s ‘structural model’ in dichotic listening.

The DL paradigm investigates perceptual and cognitive asymmetry in the auditory system. It was first implemented by Kimura. ‘Dichotic’ means ‘listen to two different signals at the same time’; one in the left ear, the other in the right ear (Kimura 1961a, 1961b). In normal hearing, the right ear sounds reach the left auditory cortex, and the sounds received from the left ear reach the right auditory cortex. Information from the right ear is ‘directly’ transmitted to the left auditory cortex in a crossed pathway, while the left ear’s information is only ‘indirectly’ transmitted to the left auditory cortex (via crossed pathway, right auditory cortex, and corpus callosum) (Kimura 1961a, 1967, 2011) (see Figure 1). Therefore, people with normal hearing typically score higher in the right ear than in the left ear in a non-forced (NF) condition. This difference increases with the forced right (FR) condition and decreases with the forced left (FL) condition (Hewitt 2018). In the FR condition, the instruction to report from the right ear follows the bottom-up deviation towards the right ear stimulus, resulting in increased right ear advantage (REA). The typical response model of the FR task synergistically follows the contralateral pathway of the brain. Therefore, working with stimuli in language processing is considered a bottom-up asymmetry. The task request causes the right ear score to be increased relative to the left ear score. On the other hand, the FL condition requires the participant to engage in top-down attentional control and inhibition of prepotent responses, as it involves a conflict between the bottom-up stimulus-driven processes favouring an REA and the instruction to report left ear stimuli. Also, it requires strategies for executive cognitive control (Bless et al. 2015; Hugdahl et al. 2009).

Few studies are found in patients with AIS in which auditory perceptual and cognitive asymmetry are investigated using the DL paradigm. The first of these studies was carried out by Goldberg et al. (1995), where attention was not directed to any ear. As a result of comparing patients with AIS and healthy individuals, the right ear advantage in AIS patients is higher, and it was concluded that the cerebral cortex is more asymmetrical or lateralised in patients with AIS. The second study states that patients with AIS who have progressive scoliosis in conditions where attention is not directed to any ear in the DL paradigm have less left hemisphere dominance than patients with AIS who have non-progressive scoliosis (Enslein & Chan 1987).

According to the results of these studies, it could be said that auditory perceptual and cognitive functions are affected in patients with AIS and progressive scoliosis. However, these studies performed the DL paradigm in patients with AIS, only under NF conditions. No studies have examined any ear attention conditions (FL and FL) with the DL paradigm in patients with AIS. This is attached to a top-down process which has not yet been considered. Therefore, our study investigated whether perceptual and cognitive asymmetry exists in the auditory system in patients with AIS, even when attention is directed to theright and left ears. It also aimed to examine whether the asymmetry varies according to patients’ curvature characteristics with AIS.

## Method

Individuals diagnosed as AIS referred to the Department of Orthotics and Prosthetics, School of Physical Therapy and Rehabilitation, Dokuz Eylül University, between August 2014 – May 2015 were included in our study. We included 38 patients (33 females, 5 males) with AIS and 10 healthy individuals (8 females and 2 males) as a control group.

The inclusion criteria were as follows: (1) having a diagnosis of AIS; (2) being between 10 and 16 years old; (3) having a Cobb angle of 20° to 50°; (4) a Risser sign determined to be 0–3; (4) being right-handed and being within normal limits of simple audiometric test values. The exclusion criteria were determined as: (1) previous spinal operations; (2) accompanying mental problems; (3) other neurologic, muscular, or rheumatic diseases; (4) being left-handed, and having non-idiopathic scoliosis.

**FIGURE 2:**
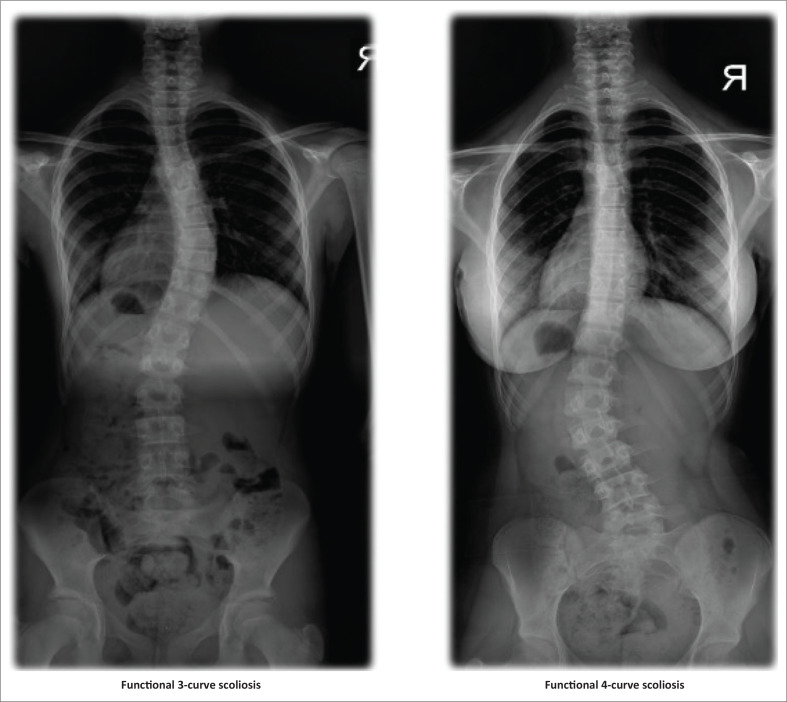
Samples of the functional 3-curve and functional 4-curve scoliosis samples according to the Lehnert-Schroth classification.

Before our study commenced, each participant underwent an anterior-posterior x-ray. The x-ray results were used to measure the scoliosis degree with the Cobb method and to evaluate the iliac crest’s growth plates’ closure with the Risser sign (Morrissy et al. 1990; Reem et al. 2009). Then Lehnert-Schroth classification was used to determine curvature patterns of patients with AIS. According to the classification, in functional 3-curve scoliosis, shoulder-neck section, thoracic section, and lumbopelvic section are flexed and distorted in the frontal, sagittal and transverse planes. In functional 4-curve scoliosis, the lumbopelvic section is divided into a lumbar section and a pelvic section (Lehnert-Schroth 2007) (see Figure 2).

### Simple audiometric test

Simple airway transmission thresholds of individuals’ left and right ear were determined with an audiometric device (Sibel Elektromedicina, S.A. Spain, model: AC 50D). When auditory hearing thresholds were determined, the evaluation was first started at 1,000 Hz and then evaluated at other frequencies. The applied frequency was reduced by five decibels (dB), until participants do not hear the sound. Individuals with a hearing threshold of 20 dB or higher for each ear and no ± 15 dB difference between the two ears were included.

### Edinburg Handedness Inventory

Edinburgh Handedness Inventory (EHI) was used to assess hand preference. The preferred hand during writing, drawing, throwing objects, using scissors, brushing teeth, using a knife, using a spoon, using a broom, burning a match, and opening a box was established (McFarland & Anderson 1980). Accordingly, the individual’s laterality coefficient (LQ) was calculated with the following equation: LQ = (ΣR-ΣL) / (ΣR + ΣL) × 100

According to the Edinburgh hand preference scores, those with a positive LQ coefficient are considered right-handed and with a negative coefficient to be left-handed (Oldfield 1971). Participants with a left-hand preference were not included, as hand preference (right and left-handed) affects auditory asymmetry (Foundas et al. 2006).

### Dichotic Listening paradigm

The DL paradigm, provides behavioural data, is an easy-to-implement and non-invasive method used in auditory system asymmetry in humans (Hiscock et al. 2000). It is also a reliable method for use in adolescents (Kelley & Littenberg 2019). The DL paradigm was applied to participants seated in an isolated room with their eyes open. In this application, / ba /, / da /, / ga /, / pa /, / ta /, / ka / syllabic combinations were used as is the case in classical dichotic applications. In terms of their suitability for Turkish society, the audio files were prepared by Dokuz Eylül University, Music Sciences Sound Studio, and were standardised by sound experts. An application developed in Matlab software and developed special hardware (Embedded Microcontroller Stimulus Unit [EMISU]) was transferred from the computer in digital Wav format (Ozgoren et al. 2009).

There were 30 different (heteronym) and 6 identical (homogeneous) combinations. In the dichotic application, 78,9 dB SPL (sound pressure level) was applied. The stimuli were transmitted in a conditioned random time interval via a programme with the prerequisite specified in the 3–6 s band. In this way, 36 pairs of dichotomous syllables were played through a Sony CDR50 type headset. Participants were asked to respond to the hearing via the digital response keyboard. The average session duration was 7.5 min, with a total of about 25–30 min. The standard DL paradigm consisted of three different conditions. In a NF condition, the participants were asked to report what they heard best. In the FR condition, attention was directed the right ear, and in the FL condition attention was directed to the left ear.

The asymmetry between the right and left ears was evaluated by calculating a ratio of correct answers in the right ear and correct responses in the left ear. The resulting value was interpreted as decreased asymmetry while approaching 1 (e.g. increased asymmetry moving away from 1).

## Statistical analysis

Statistical analysis of the data was undertaken with the ‘Statistical Package for Social Science (SPSS) for Windows version 20.0’. A Shapiro-Wilk test was used to determine the normal distribution of the data. Non-parametric tests were used in the analysis of data since the data were not normally distributed. In order to assess the difference between the two groups, a Mann Whitney U test was utilised. The *p*-value was set as (*p* < 0.05).

### Ethical considerations

The study was approved by the Dokuz Eylül University, Ethics Committee of non-interventional research in health sciences on 21 November 2013, ethics number: 2013/42-07.

## Results

The distribution of demographic characteristics and EHI values of the groups are shown in Table 1. There was no statistically significant difference between groups in terms of age, height, weight, body mass index (BMI), and EHI values (*p* > 0.05). The Cobb angle of patients with AIS was 33.94° (± 5.31). According to the Lehnert-Schroth classification, patients with AIS’s distribution was: functional 3-curve scoliosis – 14 patients, functional 4-curve scoliosis – 24 patients.

**TABLE 1:** Demographic characteristics of individuals according to groups (*n* = 48).

Variable	AIS group (*n* = 38)			Control group (*n* = 10)			*z*	*p*
	Median	Min-Max	*X*± SD	Median	Min-Max	*X* ± SD		
Age (years)	13.00	11.00–15.00	13.18 ± 1.13	15.00	11.00–16.00	13.80 ± 1.68	-1.23	0.215
Height (cm)	160.00	145.00–179.00	160.57 ± 8.06	167.00	148.00–173.00	173.00 ± 7.94	-1.58	0.112
Weight (kg)	48.00	30.00–69.00	48.05 ± 7.87	54.50	45.00–68.00	54.00 ± 10.24	-1.38	0.119
BMI (kg/m^2^)	18.40	14.00–24.00	18.59 ± 2.49	21.05	17.60–23.00	19.82 ± 2.89	-1.38	0.166
EHI	85.00	0.00–100.00	79.86 ± 22.55	80.00	50.00–100.00	75.00 ± 19.00	-1.08	0.278

*X*, Average, SD, standard deviation; AIS, adolescent idiopathic scoliosis; BMI, body mass index; EHI, Edinburg Handedness Inventory, *z*, Mann-Whitney U value; *p*, *p*-value.

In the DL paradigm, the correct responses for the right ear in the NF condition, right and left ear in the FR condition, and right ear in the FL condition did not show a significant difference between the groups (*p* > 0.05). In the NF and FL conditions, the mean number of correct responses for the left ear was significantly lower in patients with AIS than in healthy individuals (*p* < 0.05). In the NF condition, the right and left ear response rate was higher in patients with AIS than in healthy individuals; in contrast, the response rate in the left and right ears was lower in the FL condition (*p* < 0.05) (see Table 2). Also, there was no significant difference between patients with AIS with functional 3-curve and 4-curve (*p* > 0.05) (see Table 3).

**TABLE 2:** Comparison of Dichotic Listening paradigm values between the adolescent idiopathic scoliosis and control groups (*n* = 48).

Variable	AIS group (*n* = 38)			Control group (*n* = 10)			*z*	*p*
	Median	Min-Max	*X*± SD	Median	Min-Max	*X*± SD		
NF right ear	13.50	9.00–23.00	14.10 ± 3.06	13.50	3.00–18.00	11.70 ± 5.61	-0.97	0.331
NF left ear	6.50	3.00–12.00	6.71 ± 2.32	9.50	5.00–23.00	11.00 ± 5.61	-2.73	0.006**
NF right ear / left ear	2.07	1.10–7.67	2.50 ± 1.49	1.43	0.13–3.00	1.44 ± 0.92	-2.23	0.025*
FR right ear	13.00	9.00–26.00	14.02 ± 4.45	14.00	0.00–3.50	12.0 ± 7.34	-0.20	0.835
FR left ear	6.00	1.00–16.00	6.57 ± 3.29	8.00	5.00–23.00	9.11 ± 5.34	-1.45	0.148
FR right ear / left ear	2.00	0.63–25.00	3.42 ± 4.43	1.75	0.00–3.50	1.75 ± 1.23	-1.10	0.268
FL right ear	11.00	3.00–20.00	12.02 ± 4.78	11.00	1.00–18.00	10.60 ± 5.56	-0.61	0.540
FL left ear	8.50	0.001–17.00	8.15 ± 3.93	11.00	5.00–24.00	12.90 ± 5.32	-2.94	0.003**
FL left ear / right ear	0.64	0.00–5.67	0.89 ± 0.95	1.03	0.28–19.00	3.81 ± 6.38	-2.032	0.042*

*X*, Average; AIS, adolescent idiopathic scoliosis; s.d., standard deviation; *z*, Mann-Whitney U value; NF, non-forced. Without directing attention to any ear, FR, (forced-right) attention to the right ear, FL, (forced-left) attention to the left ear.

* , *p* < 0.05,

** , *p* < 0.01.

**TABLE 3:** Comparison of Dichotic Listening paradigm values between the Functional 3-curve scoliosis and Functional 4-curve scoliosis groups (*n* = 48).

Variable	Functional 3-curve scoliosis (*n* = 14)			Functional 4-curve scoliosis (*n* = 24)			*z*	*p*
	Median	Min-Max	*X* ± SD	Median	Min-Max	*X* ± SD		
NF right ear	12.50	9.00–18.00	13.35 ± 2.70	14.50	10.00–23.00	14.54 ± 3.23	-1.02	0.306
NF left ear	7.50	3.00–10.00	7.14 ± 2.34	6.00	3.00–12.00	6.45 ± 2.32	-0.94	0.344
NF right ear / left ear	1.76	1.13–4.50	2.18 ± 1.15	2.21	1.10–7.67	2.69 ± 1.67	-1.03	0.303
FR right ear	12.00	9.00–19.00	13.00 ± 3.21	13.00	9.00–26.00	14.65 ± 5.02	-0.83	0.404
FR left ear	7.50	3.00–12.00	7.07 ± 2.70	5.00	1.00–16.00	6.34 ± 3.68	-0.91	0.359
FR right ear / left ear	1.74	0.92–5.67	2.28 ± 1.53	2.25	0.63–25.00	4.11 ± 5.43	-1.08	0.280
FL right ear	14.50	3.00–18.00	13.07 ± 5.01	10.50	3.00–20.00	11.41 ± 4.64	-1.23	0.218
FL left ear	9.00	3.00–15.00	8.50 ± 3.08	8.00	0.00–17.00	7.95 ± 4.40	-0.56	0.573
FL left ear / right ear	0.63	0.18–2.25	0.80 ± 0.54	0.65	0.00–5.67	0.94 ± 1.13	-0.00	1.000

*X*, Average, SD, standard deviation; *z*, Mann-Whitney U value, NF, (non-forced) without directing attention to any ear, FR, (forced-right) attention to the right ear, FL, (forced-left) attention to the left ear.

## Discussion

We aimed to investigate the perceptual and cognitive asymmetry of the auditory system in AIS patients. Perceptual and cognitive asymmetry in the auditory system was evaluated using the DL paradigm. To date studies using the DL paradigm in patients with AIS have been done in the NF condition. Hence the directed attention to any ear condition (FR and FL) in our study is a first done in participants with AIS. We observed that there was asymmetry in NF and FL conditions, and this asymmetry was because the left ear responses of the patients with AIS were lower than the healthy individuals, whereas, in the FR condition, asymmetry was not observed. The asymmetry in patients with AIS did not change according to the curvature type.

In the DL paradigm, two linguistic stimuli were presented to both ears simultaneously. A typical finding with verbal stimuli in an average right-handed participant illustrates the REA when no special instruction regarding attention is provided (NF attention). The REA is thought to result from faster and more efficient structural pathways from the right ear to the contralateral left hemisphere’s language-dominant areas (Kimura 1961a, 1967). There are few studies on auditory perceptual and cognitive asymmetry in patients with AIS, and these studies investigated ear preference only in the NF condition. Goldberg et al. (1995) found that in DL, there was an increase in perceptual asymmetry in the presence of more REA in patients with AIS compared to healthy individuals. Enslein and Chan (1987) reported that patients with progressive AIS had less left hemisphere dominance than patients with non-progressive AIS. In our study, the Cobb angle of patients with AIS was similar to Goldberg’s study group and Enslein’s non-progressive group. In the NF condition, REA was similar in patients with AIS compared to the healthy individuals but left ear advantage (LEA) was significantly lower. The rate of asymmetry was significantly higher in patients with AIS. Goldberg et al. (1995) stated that the asymmetry is caused by the excess REA in patients with AIS. However, in contrast to Goldberg et al. (1995), we established that the asymmetry detected in the NF condition in patients with AIS was due to the low level of hearing in the left ear. Therefore, it can be proposed that patients with AIS in NF have difficulty in hearing and perceiving the stimuli from the left ear.

The FR and FL conditions are seen as attention tests and reflect different cognitive processes. In the FR condition, the instruction to report from the right ear follows the bottom-up deviation towards the right ear stimulus, resulting in increased REA. The typical response model of the FR task follows the contralateral pathway of the brain synergistically. Therefore, working with stimuli in language processing is considered a bottom-up asymmetry. The task request causes the RE score to increase on the LE score (Bless et al. 2015; Hugdahl et al. 2009). Attention to any ear condition of the DL test has been used in many diseases such as Alzheimer’s, schizophrenia, dyslexia etc., but such a study has not been undertaken in scoliosis (Bouma & Gootjes 2011; Helland et al. 2018; Hugdahl et al. 2008). In the FR condition, the mean right ear responses were observed to be higher than in the control group, while left ear responses were lower than in the control group, but this difference was not significant. Our results suggest that patients with AIS have perceptually and cognitively similar behavioural traits in directing attention to the right ear as healthy individuals, or they can overcome attention to the right ear.

On the other hand, the FL condition requires participants to engage in top-down attentional control and inhibit prepotent responses, as it involves a conflict between the bottom-up stimulus-driven processes favouring an REA, and the instruction to report left ear stimuli. Also, it requires strategies for executive cognitive control (Bless et al. 2015; Hugdahl et al. 2009). While there is no significant difference in FR in patients with Alzheimer’s and schizophrenia, there is a remarkable excess in REA values in the FL condition (Bouma & Gootjes 2011; Hugdahl et al. 2008). In our study, in the FL condition, right ear responses in patients with AIS were similar to healthy individuals, while left ear responses were significantly lower. The asymmetry seen in patients with AIS in the FL condition compared to healthy individuals can be thought to be due to their inability to direct their attention to their left ears. In addition, although perceptual and cognitive asymmetry were observed between patients with AIS and healthy individuals, it did not differ according to the curvature types of patients with AIS. These results suggest that perceptual and auditory asymmetry may not be affected by the type of scoliosis curvature.

There are a number of theories explaining the dominant right ear choice. In the ‘structural model’ of DL, Doreen Kimura (1961a) stated that the sounds heard from the right ear reach the left auditory cortex, and the sounds heard from the left ear reach the right auditory cortex using cross-ascending pathways. Kimura (1961a) suggested that right ear input is easily accessible to the left hemisphere, but that left ear input is weaker while being moved to the left hemisphere by the corpus callosum after receiving the right hemisphere. Westerhausen and Hugdahl (2008) noted that the right ear preference is derived from the functional integration of the corpus callosum as a structural and behavioural model. In patients with AIS, the corpus callosum’s morphological anomalies responsible for interhemispheric communication are reported (Domenech et al. 2011; Joly et al. 2014; Shi et al. 2009; Wang et al. 2009). Based on Kimura and Westerhausen’s theories, the corpus callosum’s structural changes in patients with AIS may be considered the reason why these patients cannot direct their attention to the left ear. However, more comprehensive studies are needed to reach this conclusion.

The limitations of our study include: the small number in the control group and that there are very few DL studies on AIS in the literature that include all the DL paradigm conditions making it difficult to compare and discuss our results.

All DL paradigm conditions of patients with AIS were evaluated and presented for the first time in our study. There is therefore a need for future studies to investigate the relationship between corpus callosum and left ear attention in patients with AIS.

## Conclusion

Perceptual and cognitive asymmetry or lateralisation in the auditory system in patients with AIS are present. We showed that the asymmetry seen in patients with AIS might be caused by the inability to direct attention to the left ear, which is not affected by the curvature type. The management of AIS should include consideration of the perceptual and cognitive asymmetries, in addition to usual conservative physiotherapy.
